# The political dimension of sexual rights. Commentary on the paper by Chandra-Mouli et al.: a never-before opportunity to strengthen investment and action on adolescent contraception, and what we must do to make full use of it

**DOI:** 10.1186/s12978-018-0458-y

**Published:** 2018-01-30

**Authors:** Leon Bijlmakers, Billie de Haas, Anny Peters

**Affiliations:** 1 Department for Health Evidence, Radboud University Medical Centre, PO Box 9101, 6500 HB Nijmegen, The Netherlands; 2 Population Research Centre, University of Groningen, PO Box 800, 9700 AV Groningen, The Netherlands; 3 Nijmegen, The Netherlands

**Keywords:** Adolescent health, Contraception, Sexual health, Sexual rights

## Abstract

**Background:**

The recent commentary article in this journal by Chandra-Mouli et al. speaks of a never-before opportunity to strengthen investment and action on adolescent contraception. We endorse the positive ‘can-do’ tone of the article, but noticed that at least four issues, which in our view are crucial, merit a comment.

**Main body:**

First of all, the article suggests that there is some sort of shared interest, based on a presumed global consensus around the use of contraceptives by adolescents – which is not the case: sexual rights are controversial. Secondly, for real progress in adolescent contraception to occur, we believe it is critical to thoroughly investigate and mention the factors, including political ones, that would need to be overcome. Thirdly, new avenues need to be explored that allow for accurate and positive teaching of adolescents about contraception in socio-cultural and political environments that are ambivalent about the issue. Fourthly, barriers at the global level that we already know of should not be silenced. There is sufficient evidence to call upon donors and international agencies to choose position and stop obstructing women’s – including young women’s – access to a broad range of contraceptives. The ‘She Decides’ movement is a heartening example.

**Conclusion:**

It is crucial to acknowledge the political dimension of sexual rights. It requires solutions not only at national levels, but also at the global level.

## Plain English summary

The recent commentary article by Chandra-Mouli and colleagues speaks of a never-before opportunity to strengthen investment and action on adolescent contraception. It calls for five things that would need to be done differently to improve access to and provision of contraceptive services to adolescents, and five things that countries would need to do to expand equitable access to quality contraceptive services for adolescents. Four issues, which in our view are crucial, merit a comment.

Firstly, sexual rights are controversial and this apparently precludes a definition at the level of the United Nations. Secondly, for real progress in adolescent contraception to occur, it appears critical to thoroughly investigate and mention the factors, including political ones, that would need to be overcome. Thirdly, we endorse the suggestion that Government-led school-based education and involvement of civil society organisations are suitable ways to support programme implementation, but the ambivalence towards adolescent sexuality requires attention. And fourthly, barriers at the global level should not be silenced, in particular the fact that certain agencies put ideology before evidence. By obstructing (young) women’s access to contraception they deliberately undermine women’s and adolescent girls’ sexual and reproductive health and rights, which negatively affects their health and wellbeing. A couple of international platforms and global movements acknowledge there barriers and combine research and advocacy in order to bring influence policy.

## Background

The recent commentary article by Chandra-Mouli et al. [1] calls for five things that would need to be done differently to improve access to and provision of contraceptive services to adolescents, and five things that countries would need to do to expand equitable access to quality contraceptive services for adolescents. The article is well referenced and sends out some very clear messages, arguing that three critical factors make this ‘never-before’ moment for action so unique. We endorse the positive ‘can-do’ tone of the article. We noticed though that at least four issues, which in our view are crucial, merit a comment.

## Sexual rights are controversial

First of all, the article suggests that there is some sort of shared interest, based on a presumed global consensus around the use of contraceptives by adolescents – which is not the case.

One of the reasons that are mentioned why adolescents are still unable to obtain and use contraceptives is that in many places laws and policies prevent the provision of contraception based on age or marital status. The underlying reasons which are not mentioned, are at least two-fold: (1) sexual rights are contested, particularly of women and minority groups; and (2) in many societies there is a general fear and anxiety surrounding adolescent sexuality.

The Human Reproduction Programme page on the website of WHO, which has been working in the area of sexual health since at least 1974, illustrates the first point. The website provides definitions of sex, sexual health and sexuality [2], but for sexual rights it does not. The webpage merely provides a working definition, with an explicit comment that “this definition does not represent an official WHO position and should not be used or quoted as such. It is offered instead as a contribution to ongoing discussion about sexual health”. The controversy about sexual rights apparently precludes a definition at the level of the United Nations.

## Political factors at the national level

Secondly, the article acknowledges the fact (on page 6) that national laws and policies vary a great deal in terms of how enabling they are; and it mentions three criteria (i.e. those identified by the New York-based Population Reference Bureau) that were found to be obstructive, particularly in the Democratic Republic of Congo and to a lesser extent in Nigeria, namely: external authorization, age restrictions and marital status restrictions. We endorse the call for implementation research that sheds light on context-specific programmatic challenges and employs methods to overcome identified obstacles. It is important to know who imposes those restrictions and for what reason; whether any efforts have been made in these countries or internationally to oppose or alleviate such restrictions, and the results of those efforts. Studies have been done that indiscriminately show the political dimensions as well as the impact of age and marital status restrictions to access sexual and reproductive health services. Yarrow et al., for instance, argue that laws that restrict children’s access to such services deny them the ability to access essential information, advice and treatment, placing them at risk [3]. As to the question why Ethiopia is doing so much better than Burkina Faso and Nigeria, where contraceptive use is stagnating or even declining, Chandra-Mouli et al. refer to Hounton et al. [4] who suggest that Ethiopia achieved its good results through a range of actions, including supportive policies for contraceptive use for adolescents, regardless of marital status or age. It is worth adding that the narrowing of the equity gap in Ethiopia was most notable for childbearing adolescents with no education or living in rural areas. For Nigeria, the paper suggests that some of the subnational variations (i.e. between states) in contraceptive use among sexually active adolescents point to possible differences in policy, strategies and investments by local governments in women’s, children’s and adolescents’ health; as well as differences in cultural and societal norms and values about keeping adolescent girls in school, curtailing child marriage and increasing access to modern contraception for all women of reproductive age. For real progress in adolescent contraception to occur, it appears critical to thoroughly investigate and mention the factors, including political ones, that would need to be overcome.

## Ambivalence requires attention

Thirdly, we endorse the suggestion by Chandra-Mouli et al. that Government-led school-based education and involvement of civil society organisations are suitable ways to support programme implementation. The authors illustrate how collaborative efforts of an indigenous nongovernmental organisation and the Indian government included developing and refreshing teacher skills, building community support and dealing with resistance, when this arose. Other studies however show the difficulties encountered by trained teachers, civil society leaders and adult role models in adolescents’ lives to actually deliver evidence-based and positive messages about contraception. De Haas for instance ([5], forthcoming), found that Ugandan teachers’ professional identity may go beyond, differ or even conflict with the required qualities for sexuality educators. In her study, some teachers acknowledged that their students were sexually active but pretended that these students were ‘sexually innocent’. Teachers took this position as a coping strategy, because acknowledging that their students were sexually active would mean that as teachers they had failed to instil the abstinence-only message as per socio-cultural norms and school regulations and to keep their students ‘morally upright’. Also, teaching contraception against school regulations could make them lose their jobs. New avenues need to be explored that allow for accurate and positive teaching of adolescents about contraception in socio-cultural and political environments that are ambivalent about adolescent sexuality. In order to address the vulnerability of sexuality educators in such challenging environments, the Dutch-based NGO Rutgers developed a manual for adopting a ‘whole-school approach’ for sexuality education. One of the five areas for action involves the creation of ‘a safe and healthy school environment’, which includes the revision of school regulations that are supportive of students’ sexual and reproductive health and rights [6].

## Barriers at the global level should not be silenced

Fourthly, the article by Chandra-Mouli et al. does not address non-adherence to international commitments. It was encouraging that the FP2020 commitments were endorsed at the London summit on family planning in 2012 by several governments, multilateral organizations, foundations, the private sector, and civil society; and that they have been re-endorsed recently (in July 2017) by an even larger group [7]. But one cannot remain silent about the fact that one of the most influential countries – which is also one of the largest bilateral donors – opposes freedom of contraceptive choice for unmarried women. And that it has re-enacted and expanded policies that are driven purely by ideology [8].

A recent review by Santelli et al. [9] found that promotion of abstinence-only-until-marriage (AOUM) policies by the US government has undermined sexuality education in the US and in US foreign aid programmes; that AOUM programmes are not effective in delaying initiation of sexual intercourse or changing sexual risk behaviours, but they continue to be funded; and that, by their design, these programmes inherently withhold young people information about human sexuality, and sometimes provide medically inaccurate and stigmatising information.

The recent paper by Pugh et al. [8] asserts that the recent re-enactment and expansion of the Mexico City Policy, widely known as the Global Gag Rule, by the Trump administration will have a dramatic impact on the funding situation of not only organisations that focus on reproductive health, but all departments and agencies that provide global health assistance. The negative implications of the Global Gag Rule extend far beyond access to safe abortion information and services, for which it was meant initially: there is little doubt that it will have negative effects on the health, well-being and empowerment of women and girls worldwide.

Chandra-Mouli et al. mention the “condoms-only mindset”, as one of the one-size fits-all approaches that must be shunned. We agree, but what about AOUM approaches? And what about the re-enactment and expansion of the Global Gag Rule? Remaining silent about the abstinence-only-until-marriage doctrine puts the claim that there is “an unprecedented moment in history” which provides a “never-before opportunity to strengthen investment and action on adolescent contraception” into a different perspective. Let us not shy away from saying that AOUM policies and programmes and the Global Gag Rule are problematic – both scientifically and ethically – as they threaten fundamental human rights to health, information, and life, especially for adolescents. With Pugh et al. we wish take a position and shout out loud that putting ideology before evidence, undermines women’s and adolescent girls’ sexual and reproductive health and rights.

Whilst we agree that opportunities exist and must be seized, let’s call a spade a spade and point out both today’s crude measures and the subtle forces that obstruct the achievement of the FP2020 goals and SDG. These are difficult times and it is more important than ever to acknowledge and celebrate the creativity, resistance and perseverance of the SRHR community and women’s rights movements. Research and advocacy, and the combination of these two, continue to be needed so as to identify and overcome the obstacles. Meanwhile, there is sufficient evidence to tackle the barriers that we already know of, especially at the global level where donors and international agencies, in spite of the rhetoric they tend to use, obstruct women’s (and thereby young women’s) access to a broad range of contraceptives, including commodities that are negatively portrayed, often in a non-purposive and implicit manner [10]. Knowledge platforms such as Share-Net International continue their fight for better SRHR outcomes by counterbalancing ideology with evidence and facilitating the translation of evidence into policy [11]. One of the heartening efforts to boost support for women’s sexual and reproductive health and rights is the ‘She Decides’ global movement. Sparked by Trump’s reinstatement of the Global Gag Rule and launched through the leadership of the Dutch Minister of Foreign Trade and Development Cooperation in early 2017, She Decides promotes, provides, protects and enhances the fundamental rights of every girl and woman [12]. Endorsed by more than 30 Governments, including Rwanda, Chad, South Africa, South Korea, Senegal, Nigeria and Mozambique, She Decides received attention at the Global Citizen pop festival in Hamburg, the FP2020 conference in London and at the United Nations in New York.

## Conclusion

It is crucial to acknowledge the political dimension of sexual rights. It requires solutions not only at national levels, which Chandra-Mouli et al. rightfully plead for, but also at the global level.
